# NTRK Fusions and TRK Inhibitors: Potential Targeted Therapies for Adult Glioblastoma

**DOI:** 10.3389/fonc.2020.593578

**Published:** 2020-11-30

**Authors:** Yuekun Wang, Piaopiao Long, Yu Wang, Wenbin Ma

**Affiliations:** Department of Neurosurgery, Peking Union Medical College Hospital, Chinese Academy of Medical Sciences and Peking Union Medical College, Beijing, China

**Keywords:** glioblastoma multiforme, tropomyosin receptor kinase inhibitor, targeted therapy, precision oncology, neurotrophic tyrosine receptor kinase fusion

## Abstract

**Introduction:**

Glioblastoma multiforme (GBM) is the most common primary central nervous (CNS) system malignancy with a poor prognosis. The standard treatment for GBM is neurosurgical resection, followed by radiochemotherapy and adjuvant temozolomide chemotherapy. Predictive biomarkers, such as methylation of the promoter region of the O6-methylguanine DNA methyltransferase (MGMT) gene, can successfully distinguish subgroups with different prognosis after temozolomide chemotherapy. Based on multiomics studies, epidermal growth factor receptor (EGFR), vascular endothelial growth factor (VEGF), BRAF V600E mutation, neurotrophic tyrosine receptor kinase (NTRK) fusions and other potential therapy targets have been found.

**Methods:**

We have reviewed the preclinical and clinical evidence for NTRK fusions and TRK inhibitors therapy in cancers with NTRK fusions in pan-cancer and gliomas.

**Results:**

Several NTRK1/2/3 fusions have been reported in GBM and preclinical studies have proven that NTRK fusions are potential driver mutations in some high-grade gliomas. Tropomyosin receptor kinase (TRK) inhibitors have shown efficacy as targeted therapies for extracranial tumors with NTRK fusions in recent clinical trials, with potential CNS tolerability and activity. However, whether NTRK gene fusions can affect survival status, the efficacy and resistance of TRK inhibitors in GBMs are lacking high-level evidences.

**Conclusions:**

For GBM patients, NTRK fusions and TRK inhibitors are potential target therapy strategy but remain biological mechanism and clinical significance unclarified. More clinical data and future clinical trials are needed to provide more evidence that supports targeted therapy for GBM with NTRK fusions.

## Introduction

Glioblastoma multiforme (GBM) is the most common primary malignant tumor of the central nervous system (CNS) and accounts for 48% of CNS malignancies, with a morbidity of 3.22 per 100,000 people. The morbidity of GBM increases positively with age, and the median age of patients is 65 years old (1). Recently, the Consortium to Inform Molecular and Practical Approaches to CNS Tumor Taxonomy (cIMPACT-NOW) recommended that GBM with IDH mutations should be referred to as IDH-mutant astrocytoma and excluded from GBM diagnosis (2). Additionally, except for conditions with pathological characteristics of microvascular proliferation or necrosis, astrocytomas with epidermal growth factor receptor (EGFR) amplification, combined whole chromosome 7 gain and whole chromosome 10 loss (+7/−10), or telomerase reverse transcriptase (TERT) promoter mutation should be diagnosed as GBM (3).

GBM is an aggressively invaded tumor with poor prognosis. The standard therapy for GBM is neurosurgical resection followed by temozolomide (TMZ) radiochemotherapy and adjuvant TMZ chemotherapy, with or without a tumor treatment field (TTF) therapy; the median overall survival (mOS) of patients with or without TTF therapy is 21 months (m) or 14.6 m, respectively (4, 5). Methylation of the promoter region of the O6-methylguanine DNA methyltransferase (MGMT) gene is a predictive biomarker of treatment response in GBM. Approximately 45% of GBM patients have a positive status of MGMT promoter methylation, and those patients have an mOS of 20–30 m under standard therapy (6, 7). Based on multiomics studies, EGFR, vascular endothelial growth factor (VEGFR), platelet-derived growth factor receptor (PDGFR), BRAF V600E mutation, histone 3 K27M (H3K27M) mutations, and neurotrophic tyrosine receptor kinase (NTRK) fusions are potential therapeutic targets in GBM, and clinical trials and clinical application are recommended in the future (8).

NTRK genes, including NTRK1/2/3, encode the Tropomyosin receptor kinase A/B/C (TrkA/B/C). During the period of embryo development, Trks regulate neurological homeostasis. TrkA/B/C have roles in pain responses, movement, body temperature and weight regulation, memory, and proprioception (9). Over-activation of Trk proteins, which is mainly regulated by the NTRK gene fusions, can lead to oncogenesis in variants types of cancer (10). NTRK fusions is a results of connection between 3’-end of Trk coding gene and 5’-end of partner gene, either through intrachromosomal or interchromosomal rearrangement. The NTRK fusions express fusion proteins with intact tyrosine kinase domain, which is the functional domain of Trk proteins, and result in persistent upregulation of downstream signal pathways (11).

NTRK fusions are detected at a relatively low rate in most types of cancer, with an average rate of 0.31% in adults and children. However, NTRK fusions can be detected in over 80% of cases of infantile fibrosarcoma and secretory breast carcinoma (12). NTRK fusions were first found to be driver mutations in colorectal cancer (13). In glioma, several preclinical research indicated that NTRK fusions were a potential driver mutations in glioma (14). Also, in clinical case, low-grade glioma with NTRK fusions and no other classical driver mutation shown further malignant tumor behavior, which mimic high-grade glioma (15). Recently, clinical trials indicated that Tropomyosin receptor kinase (TRK) inhibitors had potential tolerability and activity in multiple cancers and CNS tumors (16).

## Identifying NTRK Fusions in GBM

Based on The Cancer Genome Atlas (TCGA) database and previous studies, NTRK fusions are detected in approximately 0.56–1.69% of adult GBM patients; this detection rate is similar to that in adult low-grade glioma but is much lower than that in pediatric high-grade glioma. For children younger than 3 years old who are diagnosed with high-grade glioma, the detection rate of NTRK fusions ranges from 5.3 to 40% (9, 17). In other common types of adult cancer, the detection rate of NTRK fusions in lung, breast cancer, colorectal cancer are 0.18, 0.18, and 0.97% respectively through TCGA database, similarly to GBM (9).

The NTRK fusions that have been reported in GBM are shown in **Table 1**. In 2013, 2 studies separately reported NTRK fusions in GBM for the first time. Shah et al. reported BCAN-NTRK1 and NFASC-NTRK1 fusions through RNA sequencing of patient samples from two centers with a detection rate of 0.5% (18), and Frattini et al. simultaneously found the same result in the TCGA database (20). Later, Kim et al. and Stransky et al. validated these results (19, 22). Zheng et al. reported ARHGEF2-NTRK1 and CHTOP-NTRK1 fusion through reverse transcription-polymerase chain reaction (RT-PCR), and no similar conclusions have been reported to date (23). In 2018, Ferguson et al. and Gatalica et al. reported NTRK 2/3 gene fusions in adult GBM (17, 26), and most recently, Torre et al. and Woo et al. identified several new NTRK fusions (**Table 1**) (21, 27). However, as molecular pathology, such as MGMT promoter methylation and EGFR mutations, was acknowledged to playing an important role in the management of adult GBM, more studies are need to clarify the status of NTRK fusions in patients with different subtype of GBM.

**Table 1:** NTRK fusions identified in GBM.

Gene	Partner gene	Location of partner gene	Frequency	Reference
NTRK1	BCAN	1q23.1	1/185 1/162 1/139 N/A	Shah et al. (18) Kim et al. (19) Frattini et al. (20) Torre et al. (21)
	NFASC	1q32.1	1/185 1/162 1/139 1/157	Shah et al. (18) Kim et al. (19) Frattini et al. (20) Stransky et al. (22)
	ARHGEF2	1q22	Un N/A	Zheng et al. (23). Torre et al. (21).
	ARHGEF11	1q23.1	N/A	Torre et al. (21)
	CHTOP	1q21.3	Un N/A	Zheng et al. (23) Torre et al. (21)
	AFAP1	4p16.1	N/A N/A	Rosen et al. (24) Hechtman et al. (25)
	TPM3	1q21.3	N/A	Rosen et al. (24)
	MEF2D	1q22	1/982*	Gatalica et al. (26)
	CD247	1q24.2	N/A	Torre et al. (21)
	LMNA	1q22	N/A	Torre et al. (21)
	TPM3	1q21.3	N/A	Torre et al. (21)
NTRK2	GKAP	9q21.32	1/219 1/982*	Ferguson et al. (17) Gatalica et al. (26)
	KCTD8	4p13	1/219 1/982*	Ferguson et al. (17) Gatalica et al. (26)
	TBC1D2	9q22.33	1/219 1/982* N/A	Ferguson et al (17) Gatalica et al. (26) Hechtman et al. (25)
	BCR	22q11.23	N/A 2/982* N/A	Rosen et al. (24) Gatalica et al. (26) Torre et al. (21).
	PRKAR2A	3p21.31	1/982*	Gatalica et al. (26)
	PAIP1		N/A	Torre et al. (21).
NTRK3	EML4	2p21	1/219 N/A N/A	Ferguson et al. (17) Rosen et al. (24) Hechtman et al. (25)
	ZNF710	15q26.1	N/A	Hechtman et al. (25)
	CEMIP (KIAA1199)	15q25.1	N/A	Torre et al. (21)
	DLG1	3q29	N/A	Torre et al. (21)

Un: unknown, Zheng et al. conduct a study with unknown number of patients included with GBM.

N/A: not applicable, Rosen et al., Hechtman et al. and Torre et al. include patients with confirmed NTRK fusions.

*Gatalica et al. include 982 patients with glioma and number of patients with GBM is not reported.

An ETV6-NTRK3 fusion has been reported in many types of solid cancers, including secretory breast carcinoma, carcinoma of salivary glands and gastrointestinal system, and acute myelocytic leukemia (AML) (9). ETV6-NTRK3 was also detected at a high frequency in infantile high-grade gliomas but was rarely detected in adult GBM (28). Gatalica et al. reported a patient of GBM unknown age with an ETV6-NTRK3 fusion (26).

## Molecular Biology of NTRK Fusion-Targeting Therapy and Resistance

**Figure 1:**
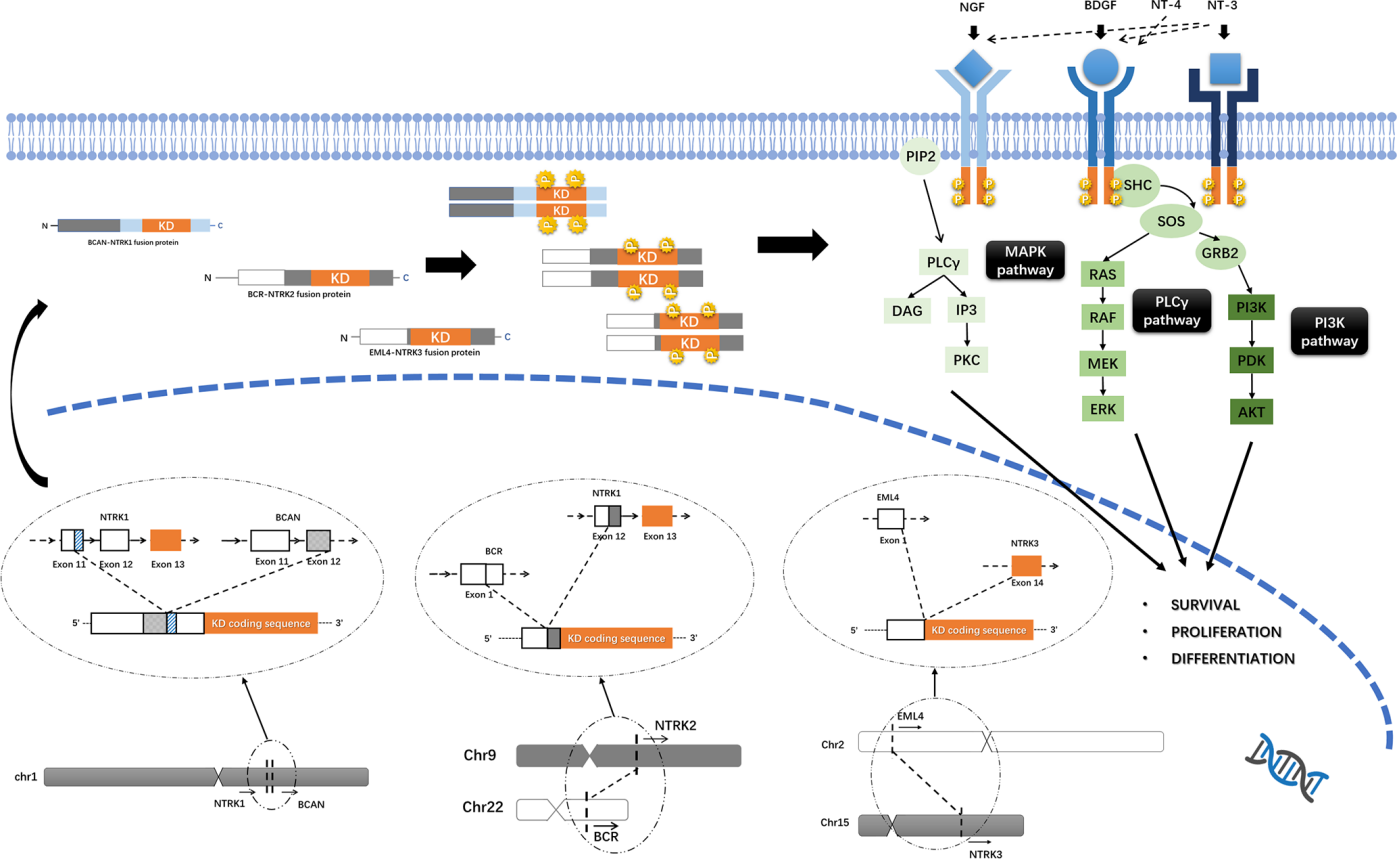
Trks pathway and the oncogenic mechanism of NTRK fusions. Trk proteins contain an intracellular tyrosine kinase domain and promote cell proliferation and survival through downstream MAPK/ERK, PLCγ/PKC and PI3K/AKT pathways. Functional Trk-fusion proteins contain a complete tyrosine kinase domain, and the partner gene is expressed in a homodimer, which induces ligand-independent activation of the tyrosine kinase domain and upregulates downstream cancer-associated pathways. The mechanism of NTRK fusions, for example, BCAN-NTRK1 (19), BCR-NTRK2 (15), and EML4-NTRK3 (25) gene fusions formation was shown.

NTRK1/2/3 encode TrkA/B/C of the TRK family, respectively. Trk proteins contain an extracellular ligand-combining domain, a transmembrane domain and an intracellular tyrosine kinase domain. TrkA/B/C are regulated by nerve growth factor (NGF), brain-derived growth factor (BDGF) and neurotrophin-4/5 (NT-4/5) and NT-3, respectively, and promote cell proliferation and survival through downstream mitogen-activated protein kinase/extracellular regulated protein kinases (MAPK/ERK), phospholipase C gamma/protein kinase C (PLCγ/PKC), and phosphatidylinositol 3 kinase/protein kinase B (PI3K/AKT) signaling (**Figure 1**) (29).

NTRK1 is located on 1q21-22, while NTRK2 is located on 9q21.1. Both NTRK1/2 genes have 17 exons with exons 13–17 encoding the tyrosine kinase domain. Similarly, the NTRK3 gene is located on 15q25.3 and has 18 exons, with exons 13-18 encoding the tyrosine kinase domain. Generally, NTRK fusions occur in introns, with the 5’-region of the fusion partner gene connected with the 3’-end of the NTRK gene. For NTRK1/2/3, the gene breakpoint is located at the 5’ region to exons 13–17 (and exons 13–18 for NTRK3), leaving a complete sequence that encodes the tyrosine kinase domain (11, 30). Approximately 60% of NTRK1 fusions are intrachromosomal rearrangements, but for NTRK2/3, more than 70% of gene fusions are interchromosomal (30).

The formation of gene fusions is a complex process, including balanced rearrangements and unbalanced rearrangements in chromosomal level, generally. Balanced chromosomal rearrangements, including translocation, insertion and inversion, result in no loss or gain of genes. And unbalanced chromosomal rearrangement, such as gene deletion, can lead to copy number variations. In tumor cell, structural variation maybe more complex and usually involved more than two genes, such as local-distant cluster, chromoplexy or chromothripsis, which may cause gene fusions. Also, transcripts can fusion together by trans-splicing and cis-splicing and produce a functional fusion protein without chromosomal rearrangement (31–33). Through gene-sequencing technique, gene breakpoint in any exons or introns were located in several studies (15, 19, 25); however, the structural variation of genetic materials that produce NTRK fusions remains unclear.

Most functional NTRK fusion proteins lack the extracellular ligand-combining domain of Trk; instead, the partner gene is expressed in a homogenous dimer, which induces ligand-independent activation of the tyrosine kinase domain and upregulates downstream cancer-associated pathways. However, some NTRK fusions cannot be expressed into functional proteins, and the mechanisms remain unclear (11, 30). NTRK fusion mutation may lead to a change of methylation status of partner genes, and GBM with NTRK fusions shown different gene methylation pattern, which is needed further researches in the future (21). Also, the relationship between the detecting frequency, variants and biological mechanisms of NTRK fusions with typical molecular pathology in adult glioma, such as IDH mutation, EGFR amplification, MGMT promoter methylation and so on, are remain unknown.

NTRK genes are rarely expressed in GBM, but a significantly higher level of expression of NTRK1 and increased activation of the NGF/TrkA pathway were found in GBM with NTRK1 fusions (18, 19). However, NTRK fusions were proved to be a potential driver mutation in glioma. Functional NTRK fusions lead to an activation of Trk proteins, which contributed to the development of glioma (34).Preclinical research indicates that neural stem cells(NSCs) can progress into high-grade glioma when the BCAN-NTRK1 fusion protein is expressed artificially by CRISPR-Cas9 genome editing tools (14). Also, another team transduced BCAN-NTRK1 gene fusions into NSCs through CRISPR-Cas9 system and replication-competent avian leukosis virus splice acceptor-tumor virus (ARCAS-TVA) system, and intracranial injection of those NSCs into mice leads to a development of high grade glioma (35). As a clinical evidence, Jones et al. reported a 52-year-old patient who was pathologically diagnosed with low-grade glioma and had a BCR-NTRK2 fusion, with no IDH1/2, EGFR, or BRAF gene mutations and no codeletion of chromosomes 1p and 19q. In this case, the tumor was unusually aggressive and showed a clinical process mimicking high-grade glioma, and the patient was died with an OS of 13.5 m (15). Although specific NTRK gene fusions are shown to have oncogenesis function in several studies with animal models, further pre-clinical and clinical researches are needed to prove the role of NTRK fusions in tumorgenesis and tumor progression and clarify the mechanisms due to the lacking of prognostic value of NTRK fusions and relatively lower occurrence.

For tumors with NTRK fusions, the coactivation of Trk and other signaling pathways, such as TP53, tyrosine kinases, and cell cycle pathways, has been reported (12). The coexistence of NTRK fusions and IGF1R, CDKN2B, and CDK4 gene mutations, which can lead to TRK inhibitor resistance, has been found in lung cancer and others (36). However, few cases have been reported to have both NTRK fusions and important driver mutations, such as EGFR, ALK, and ROS1 (24). Additionally, treatment with TRK inhibitors can induce the overactivation of cancer-associated pathways, such as the MAPK pathway, and the emergence of hot-spot mutations, MET amplification, KRAS mutation, and BRAF V600E mutation, resulting in treatment failure (37). These findings suggest a new landscape of combination therapy in the future.

Aside from gene fusion, other alterations of the NTRK gene have been reported, such as somatic mutations, insertions, deletions and amplifications. Whether these mutations can act as driver mutations of cancer is still unknown. Several NTRK gene mutations can cause resistance to TRK inhibitors, including resolvent-front alteration of TRKA G595R and TRKC G623 in the ATP binding pocket of the kinase domain; TRKA G667C, TRKB G709C, and TRKC G696A alteration in the xDFG site; and gatekeeper mutations such as TRKA F589L, TRKB F633L, and TRKC F617L (38).

## Diagnostic Strategies for NTRK Fusions in GBM

The detection of NTRK fusions mainly relies on immunohistochemistry (IHC), fluorescence *in situ* hybridization (FISH), RT-PCR and next-generation sequencing (NGS). The detection of gene fusions by IHC occurs at the protein level; thus, nonexpressing NTRK fusions cannot be detected. IHC can present false-negative results in samples from both adults and children, and the sensitivity and specificity of detection of NTRK3 fusions are much lower than those for NTRK1/2 (25, 39). Naturally, TrkA/B/C proteins are expressed in adult neural tissue, and IHC with TrkA/B-specific antibodies can yield a weakly positive result in GBM (40), which complicates the interpretation of NTRK fusion results. Also, the occurrence of NTRK fusions in adult GBM is relatively lower and limited evidence supports the necessity of NTRK fusion detection by IHC in GBM (30).

FISH is the main technique for the detection of chromosomal rearrangement, but it cannot identify the 5’-ending partner gene and expression of NTRK fusions (41). Break-apart probes that target the NTRK1/2/3 gene or fused probes that are used for specific gene fusions, such as ETV6-NTRK3 fusion, are all used in the detection of NTRK fusion by FISH (42). The interpretation of NTRK fusions by FISH depends on extensive experience. Therefore, FISH is recommended for the detection of frequent NTRK fusions, especially the ETV6-NTRK3 fusion, but is not designed to be a screening test (9).

Nucleoside-based methods for detection of gene fusions, such as RT-PCR, RNA sequencing (RNA-seq) and DNA sequencing (DNA-seq), can screen known fusions and unknown NTRK fusions with a clear identification of 5’-ending partner genes. RT-PCR and Sanger sequencing can help confirm NTRK fusions that are screened by RNA-seq; this process was described in a study by Frattini et al., in which GBMs with FASC-NTRK1 fusion were detected (20). Because of the instability of RNA, false-negative results can occur in samples with degraded RNA. A study of infantile fibrosarcoma indicated that 48% of samples did not meet the quality standards for RNA-seq (42). Murphy et al. introduced a two-step diagnostic test for the detection of specific NTRK fusions: A screening test by IHC with pan-TRK antibody was performed first, followed by anchored multiplex PCR (AMP) RNA-seq to confirm the detected NTRK fusions. Compared with RNA-seq alone, the two-step diagnostic test improved the positive detection rate by 4–9% (43). In clinical trials including TRK inhibitors, DNA-seq is routinely used to detect NTRK fusions (44, 45). However, the length of the intron in the DNA sequence limits the efficacy of DNA-seq, especially in regard to NTRK2/3 fusions (10, 39). DNA-seq cannot distinguish between functional gene fusions, and other tests at the transcriptomic or protein level, such as IHC, are needed for confirmation. Hechtma et al. showed that in 23 samples with gene fusions detected by DNA-seq, two of them did not actively transcribe, including the ZNF7-NTRK3 fusion (n = 1) (25). Few studies support RT-PCR as a screening test, but NGS techniques, whose efficacy has been verified, are recommended for detecting NTRK fusions and guiding TRK inhibitor treatment (9).

Recently, molecular pathology is routinely performed to help treatment decisions. As adult GBM lead to a worse survival probability, detection of NTRK fusions shown a new chance for them, especially those with poor diagnosis under standard therapy. It is a worthy practice point to detecting NTRK fusions for GBM patients who do not have typical therapy targets and driver mutations like methelytion of MGMT promoter. The advantages and disadvantages of NTRK fusions detection assays were summarized in **Table 2**. Briefly, ICH is a rapid and widespread assay but maybe not optimal for detection of NTRK fusions in GBM due to expression of Trk proteins in GBM tissue and lower prevalence of NTRK gene fusions. FISH is the gold standard in fusion gene detection and widely utilized in clinical practice. However, it is difficult to detecting multiple targets, which is an urgent requirement recently, by FISH. Nucleoside-based methods including RT-PCR, RNA-seq, and DNA-seq can detect novel gene fusions in a high throughput way and involved in most clinical trials and pre-clinical researches, but these assays are costly, time-consuming and with extreme complexity in result interpreting and bioinformatics analysis. Further study should be done to evaluate the cost-and-effect of each diagnostic strategies of NTRK fusions in adult GBM.

**Table 2:** Advantages and disadvantages of NTRK detection assays.

	IHC	FISH	RT-PCR	NGS	
				DNA-seq	RNA-seq
Sensitivity* (25, 39, 46)	87.9–95.2%	High (High sensitivity by canonical breakpoints FISH)	High/ Variable	81.1%	Very high if RNA quality is sufficient/ Variable
Specificity* (25, 39, 46)	81.1–100%	High	High/ Variable	99.9%	Very high
Detection level	Protein	DNA	RNA	DNA	RNA
Detection of partner genes	No	No	Yes	Yes	Yes
Cost (32, 47)	Low	Low-high	Low	High	Low-High
Type of specimens (30, 47)	- PPFE; - At least 1 unstained slide	- PPFE; - At least 3 stained slides	- PPFE, frozen or stabilized tissue; - 1 mg of RNA	- PPFE, frozen tissue; - 250 ng of DNA	- PPFE, frozen or stabilized tissue; - 200 ng of RNA
Turnaround time (30, 47, 48)	1–2 days	1–3 days	5–10 days	2–4 weeks	2–4 weeks
Summary of advantages (32, 46, 49)	- Rapid, inexpensive and available easily;	- Gold standard; - Rapid; - Shown location of the gene fusions in tumor cell; - Detection of gene fusions without knowing fusion partners by breakapart FISH; - Detection several targets in a single sample by several fluorophores;	- Inexpensive; - Identification of specific gene fusion partners; - Detection at transcriptome level;	- Detection of novel fusions and identification of gene partners; - Detection of more than 1 gene fusions and multiple therapy targets simultaneously;	- Detection at transcriptome level and fusion transcripts caused by alternative splicing; - Detection of novel fusions and identification of gene partners; - Detection of more than 1 gene fusions and mutilple therapy targets simultaneously; - Focus on coding sequences but not introns;
Summary of disadvantages (32, 46, 49)	- Cannot differentiate expression of NTRK fusions and wild-type Trk proteins and needed additional confirmatory test; - Possible false negatives for detection of NTRK3 gene fusions;	- Not widespread availability; - Possible false positive for complex chromosomal translocation; - Each fusion partner needed different probes, and separated tests are needed for NTRK1/2/3 if unknown fusions; - Cannot detect functional fusion proteins; - Not a high throughput assay;	- Not widespread availability; - Target sequences of gene fusions must be known; - Difficulty in primers design for each gene fusion;	- Expensive; - Detection of gene fusions depends on platforms and limited in gene with large introns; - Difficulty in results interpreting and bioinformatics analysis;	- Expensive; - Depend on RNA quality of specimens; - Difficulty in results interpreting and bioinformatics analysis;

IHC Immunohistochemistry; FISH Fluorescence in situ hybridization; RT-PCR Reverse transcription-polymerase chain reaction; NGS Next-generation sequencing; RNA-seq RNA sequencing; DNA-seq DNA sequencing;

*sensitivity and specificity were evaluated in populations of pan-cancer;

## Target Therapy by TRK Inhibitors

TRK inhibitors that can target TrkA/B/C have potential efficacy in tumors with functional NTRK fusions, namely, those generating proteins with complete kinase domains. These inhibitors include the selective TRK inhibitor larotrectinib, the multikinase inhibitor entrectinib and others that have entered into preclinical or clinical research (**Table 3**). TRK inhibitors are recommended as a part of the initial treatment for tumors with NTRK fusions (9).

**Table 3:** Ongoing clinical trials of TRK inhibitors as target therapy to NTRK fusions.

TRK inhibitors	Targets	Registration ID	Phase	Participants
Larotrectinib (LOXO-101)	TRKA/B/C	NCT02465060	II	Adults Advanced/recurrent malignant solid neoplasm (including glioma)
		NCT04142437	–	Adults/children Locally advanced or metastatic solid tumor [including adult patients with primary central nervous system (CNS) cancer]
Entrectinib (RXDX-101/NMS-E628)	TRKA/B/C, ROS1, ALK	NCT02568267	II	Adult Solid tumor (including brain metastases)
		NCT03994796	II	Adult Brain metastases
Repotrectinib (TPX-0005)	TRKA/B/C, ROS1, ALK	NCT04094610	I/II	Young adult/children Primary CNS tumors, locally advanced/metastatic solid Tumors, lymphoma
		NCT03093116	I/II	Un Locally advanced/metastatic solid tumors (including primary CNS tumors)
Altiratinib (DCC-2701)	TRKA/B/C, MET, VEGFR2, TIE2	NCT02228811	I	Adult Locally advanced/metastatic solid tumors with MET/TRK genomic alterations
Sitravatinib (MGCD516)	TRKA/B, MET, AXL, VEGFR, PDGFR	NCT02219711	I	Un Advanced cancer
DS-6051b	TRKA/B/C, ROS1	NCT02675491	I	Adult Advanced solid tumors
		NCT02279433	I	Adult Solid tumors
PLX7486	TRKA/B/C,CSF1R	NCT01804530	I	Adult Solid tumors, tenosynovial giant cell tumor
F17752	TRK, ALK, ROS1	(EudraCT) 2013-003009-24	I/II	Adult Solid tumor
Cabozantinib (XL184)	TRK, MET, RET, VEGFR, KIT, FLT3, AXL	NCT04116541	II	Adult Solid tumor
		NCT01822522	I	Un Advanced/metastatic/unresectable solid neoplasm; HIV infection
Merestinib (LY2801653)	TRKA/B/C, MET, FLT3, ROS1	NCT02920996	II	Adult Solid tumor
Selitrectinib (LOXO-195)	TRKA, B, and C	NCT03215511	I/II	Adults/children Solid tumors
Belizatinib (TSR-011)	TRKA/B/C, ALK	(EudraCT) 2013-000686-37	I/IIa	Adults Solid tumors; lymphoma
Dovitinib	TRK, KIT, FLT3, FGFR, VEGFR	NCT01831726	II	Adults Solid tumors
ONO-7579	TRK	NCT03182257 (stopped for commercial reasons)	I	Adults Solid tumors

Un, unknown.

Clinical trials of TRK inhibitors in pan-cancer and CNS tumors with NTRK fusions are listed, and clinical trials that are designed for specific non-CNS tumors are excluded.

In 2018, larotrectinib, or LOXO-101, became the first Food and Drug Administration (FDA)-approved pan-TRK inhibitor for adult and pediatric solid tumors with NTRK fusions in the United States (50). The efficacy of larotrectinib was initially confirmed in a phase I clinical trial of soft-tissue sarcoma (51). Hong et al. performed a pooled analysis of 3 clinical trials, namely, a phase I trial in adults, a phase I/II trial in children, and a phase II trial in adolescents and adults. All patients involved were diagnosed with non-CNS solid tumors, and the majority had NTRK1/3 fusions. This pooled analysis showed an objective response rate (ORR) of 79%, and 16% (n = 24/153) of patients achieved complete response (CR). The main side effects of larotrectinib are increased alanine aminotransferase, anemia, and decreased neutrophil count, and the possibility of grade 3 or 4 side effects is lower than 3% (52). Rosen et al. reported two cases of brain metastases with NTRK fusions treated with larotrectinib, which supports the potential CNS activity of larotrectinib (53). At the annual conference of the American Society of Clinical Oncology (ASCO) in 2019, Drilon et al. presented 9 cases of CNS primary tumors (including 2 GBMs) from 2 clinical trials. Excluding 1 patient who could not be evaluated, 1 patient achieved a partial response (PR), and the other 7 patients were evaluated as having stable disease (SD) (54). The clinical trials involving larotrectinib are mainly for advanced or metastatic malignancy, and little evidence supports its efficacy in early or locally progressed tumors. A phase I clinical trial in children with locally advanced sarcomas and NTRK fusions showed the potential efficacy of larotrectinib as neoadjuvant therapy (55). Additionally, Ziegler et al. reported a pediatric recurrent high-grade glioma after initial treatment with neurosurgical resection and 13 cycles of chemotherapy. This patient underwent debulking surgery and radiotherapy after recurrent glioma was diagnosed and showed rapid progression. This patient had a detected ETV6-NTRK3 fusion and was treated with larotrectinib and showed constant remission at the 9-m follow-up without serious side effects (56).

Entrectinib, also known as RXDX-101 or NMS-E628, was approved by the FDA in 2019, 1 year after larotrectinib; this compound targets solid tumors with NTRK fusions and adult metastatic lung cancers with ROS1 rearrangement (57). A combined analysis of 2 phase I clinical trials of ALKA-372-001 and STARTRK-1 patients with NTRK fusions and no history of treatment with ROS1 inhibitors or ALK inhibitors showed a response to entrectinib. This study reported 1 patient with glioneuronal tumor and BCAN-NTRK1 fusion and 1 patient with brain metastases from lung cancer and SQSTM1-NTRK1 fusion, and both patients showed an evaluable response to entrectinib, which supports the CNS activity of entrectinib (45). Doebele et al. integrated the results of these two trials with another two clinical trials, STARTRK-2 and STARTRK-NG, and showed an ORR of 57% (n = 31/54). The major grade 3 to 4 side effects of entrectinib were weight gain and anemia, and 4% of patients showed neurological symptoms (58). Another preclinical study indicated the efficacy of entrectinib in GBM in an intracranial *in situ* high-grade glioma mouse model with BCAN-NTRK1 fusions (14).

Secondary mutations in the Trk proteins kinase domain after treatment with TRK inhibitors can induce resistance to larotrectinib or entrectinib, such as TRKA G595R, TRKB G639R, TRKC G623R and TRKC G623E variations (59). Selitrectinib (LOXO-195) and repotrectinib (TPX-0005) have proven efficacy on tumors with those variations in animal experiments (60, 61). Phase I/II clinical trials of selitrectinib and repotrectinib are ongoing (NCT03215511 and NCT03093116). Merestinib showed an antitumor effect on *in vivo* cancer models with NTRK fusions and TRKA G667C variation in a preclinical study, and a clinical trial that includes lung cancer mainly and other solid tumors is ongoing (NCT02920996) (62).

Cabozantinib (XL184) is a multitarget inhibitor and has been researched in a phase II single-armed trial in relapsed glioblastoma. For patients without a history of anti-angiogenesis therapy, high/low dose cabozantinib (140 or 100 mg/d) showed ORRs of 17.6 and 14.5% and mOS rates of 7.7 and 10.4 m, respectively (63). For GBM patients who progress after anti-angiogenesis therapy, cabozantinib is related to an ORR of 4.3% and an mOS of 4.6 m (64). This trial proved the CNS antitumor efficacy of cabozantinib, and a preclinical study indicated the function of targeting NTRK fusions; however, cabozantinib resistance is found in tumors with TKA L564H, D679G, G595R and G595L mutations (36). Currently, two clinical trials are undergoing active recruitment of patients with advanced and metastatic cancer (NCT04116541) and progressed non-small-cell lung cancer (NSCLC) (NCT01639508). Crizotinib and ponatinib are multitarget TRK inhibitors that have entered clinical trials in newly diagnosed GBM (nGBM) and recurrent GBM patients (NCT02270034 and NCT02478164), but the patients involved have unknown NTRK fusion status (9, 65). TSR-011 showed acceptable safety and toxicity profiles in a phase I trial in NSCLC, and its antitumor function remains to be elucidated in further trials (66). Milciclib is entering clinical trials with CNS tumors as an excluded criterion, but CNS activity is expected to be evaluated in the future (67). Other ongoing clinical trials involving TRK inhibitors that target NTRK fusions in pan-cancers and CNS tumors are shown in **Table 3**.

For GBM, therapies targeting NTRK fusions need further study. Rosen et al. reported a single-center cohort analysis of TRK inhibitors in multiple cancers, including salivary carcinoma and thyroid carcinoma and sarcoma, and showed an ORR of 67.6% (n = 23/34). Four patients with GBM were involved in this study, with NTRK1-AFAP1, NTRK1-TPM3, NTRK2-BCR and NTRK4-EML4 fusions, and all patients had a history of neurosurgery, radiotherapy, and adjuvant chemotherapy. However, the two evaluable patients showed no response to TRK inhibitors (24).

## Summary

With the confirmation of diagnostic and predictive biomarkers, such as IDH1/2 mutation and MGMT gene promoter methylation, molecular pathology is routinely performed to diagnose adult GBM and guides prognosis evaluation and treatment decisions. However, patients with GBMs are still suffering from a poor prognosis. For patients that accord with including criteria, clinical trials are recommended by National Comprehensive Cancer Network (NCCN) guideline in central nervous system cancers (68), which is aimed to find more efficient targets and therapy strategies. Recently, variant NTRK fusion mutations were detected in adult GBM, and NTRK fusions were shown to be potential driver mutations and therapy targets in GBM. Although the detection rate of NTRK fusions in GBM is relatively low, TRK inhibitors, which target NTRK fusions, have shown potential efficacy in CNS tumors and adult GBM in preclinical studies and clinical trials. Considering unknown prognostic value of NTRK fusions and lacking of experience in resistance to TRK inhibitors, more clinical researches are needed to evaluation the function of NTRK gene fusions in GBM and the decision tree of detection and target therapy of NTRK fusions.

Coactivation of NTRK fusions and tyrosine kinase family members, cell cycle or other cancer-associated pathways, and driver mutations indicates a new landscape of combined target therapy in GBM (12). Additionally, multiomics studies in GBM have reported several therapy targets, such as EGFR variation, BRAF mutation and NTRK fusions, and multi-arm trials that include molecular biomarkers are recommended in the design of a high-efficiency clinical trial (8, 69).

## Author Contributions

Study design: YW and WM. Manuscript writing: YKW and PL. Papers searching: YKW and PL. Manuscript formatting and revising: YKW, PL, YW and WM. All authors contributed to the article and approved the submitted version.

## Funding

1. Beijing Municipal Science and Technology Commission (7202150), 2. Beijing Municipal Science and Technology Commission [19JCZDJC64200(Z)], 3. Chinese Academy of Medical Sciences (2016–I2M–2–001), and 4. Peking Union Medical College Hospital (2019ZLH101).

## Conflict of Interest

The authors declare that the research was conducted in the absence of any commercial or financial relationships that could be construed as a potential conflict of interest.
